# A study on the effects of combined DBT and STEPPS interventions for adolescents with non-suicidal self-injury: a randomized controlled trial

**DOI:** 10.3389/fpubh.2026.1828667

**Published:** 2026-06-05

**Authors:** Suhong Wang, Yue Zhou, Yangyang Yu, Junlei Zhang, Xueli Zhu, Kaili Zhang, Ziyang Ji, Yuchun Li, Fang Yan, Chuansheng Wang

**Affiliations:** 1 The Second Affiliated Hospital of Henan Medical University, Xinxiang, Henan, China; 2 Henan Collaborative Innovation Center of Prevention and Treatment of Mental Disorder, Xinxiang, Henan, China

**Keywords:** adolescents, dialectical behavior therapy, emotion regulation, non-suicidal self-injury, systems training for emotional predictability and problem solving

## Abstract

**Objective:**

To evaluate whether an integrated Dialectical Behavior Therapy and Systems Training for Emotional Predictability and Problem Solving (DBT + STEPPS) programme is more effective than DBT alone or conventional treatment in reducing non-suicidal self-injury (NSSI) and improving emotion regulation, anxiety, and depressive symptoms among adolescents with depression and NSSI.

**Methods:**

This study recruited 192 adolescent patients with non-suicidal self-injury between March 2024 and February 2025, and they were randomly divided into the Control Group (conventional intervention), Intervention Group A (DBT intervention), and Intervention Group B (DBT combined with STEPPS intervention). Patients’ self-harm behaviors, emotional regulation strategy capabilities, and severity of illness were assessed both prior to intervention and within 48 h post-intervention. Data analysis employed one-way analysis of variance (ANOVA), Kruskal–Wallis H test, *χ*^2^ test, or Fisher’s exact test.

**Results:**

Intervention Group B exhibited lower NSSI behavior scores than both Intervention Group A and the control group, while Intervention Group A scored lower than the control group (*p* < 0.05). Regarding emotional regulation capacity, Intervention Group B demonstrated the highest adaptive strategy scores and the lowest maladaptive strategy scores (*p* < 0.05). This group also recorded the lowest anxiety and depression symptom scores (*p* < 0.05). All indicators for Intervention Group A were superior to those of the control group (*p* < 0.05).

**Conclusion:**

DBT combined with STEPPS effectively reduces NSSI behaviors, improves emotion regulation, and alleviates anxiety and depression in adolescent non-suicidal self-injurious patients. And the effect of the combined intervention is better than that of the single intervention.

**Clinical trial registration:**

This study was registered with the Chinese Clinical Trial Registry (https://www.chictr.org.cn/; Registration number: ChiCTR2100043855) on 4 March 2021. The first subject was recruited on February 13, 2025.

## Background

Non-suicidal self-injury (NSSI) refers to the deliberate, direct, and repetitive harming of one’s own bodily tissue without suicidal intent. It manifests in diverse forms, including but not limited to cutting, scratching, striking, burning, pinching, and deliberately preventing wound healing (1). Global epidemiological data indicate that the overall prevalence of NSSI among adolescents in community settings is approximately 19.3% (2), and the prevalence of NSSI significantly increases in mental health institutions. A study in China suggest that the rate can be as high as 27.4 per cent (3). Among adolescents with depression, a meta-analysis reported a prevalence of NSSI as high as 51%, with female patients exhibiting a significantly higher incidence (36%) than males (18%) (2). Moreover, this phenomenon is particularly pronounced in clinical settings, underscoring the urgency of prioritizing this group for intervention. NSSI not only threatens the physical health of adolescents, but is often accompanied by mood disorders and impaired social functioning. This urgently requires targeted intervention strategies.

In current clinical interventions for NSSI, psychological interventions predominate, with pharmacological treatments serving a supplementary role. Given the complexity of the multidimensional issues—such as emotional, cognitive, and interpersonal difficulties—often co-occurring with NSSI, many conventional psychological interventions (such as single-modality supportive psychotherapy or cognitive behavioral therapy) demonstrate certain limitations. These approaches typically rely on standardized intervention modules. While possessing universal applicability, they often fall short in dynamically adapting to each patient’s unique combination of problems, symptom severity, and contextual factors. Consequently, their efficacy is constrained, failing to meet the individualized psychological intervention needs of patients (4).

Dialectical behavior therapy (DBT) represents a novel form of psychological intervention. Evolving from cognitive behavioral therapy (CBT), it incorporates principles from Eastern philosophy and Buddhist Zen teachings, emphasizing the dialectical unity of acceptance and change. It advocates a more structured and individualized therapeutic framework involving mindfulness, emotion regulation, distress tolerance, and interpersonal effectiveness training to achieve behavioral correction and emotional management (5). DBT has been demonstrated to effectively alleviate symptoms in patients with borderline personality disorder (6) and post-traumatic stress disorder (7). It has also been preliminarily applied in psychological interventions for adolescent patients with non-suicidal self-injury (NSSI). Meta-analyses indicate that DBT helps reduce NSSI behaviors and ameliorate depressive symptoms (8, 9).

DBT is among the most empirically supported psychotherapies for adolescent NSSI, but its full implementation can be relatively intensive and is primarily centred on individual skills acquisition, crisis management, and therapist-guided behavioral change (10). By contrast, STEPPS is a brief, manualized, group-based adjunctive intervention that also targets emotion dysregulation, but places greater emphasis on simplified psychoeducation, repeated rehearsal of emotion-management strategies, structured problem solving, and involvement of the family or broader support system (11). Moreover, the STEPPS has demonstrated reliable application in interventions targeting self-harm behaviors associated with borderline personality disorder (11).

Family involvement is not unique to STEPPS and is already recognized in DBT-based interventions for adolescents and their caregivers. In particular, DBT-A commonly includes parental participation, and Family Connections (12) is an established DBT-based, manualized programme developed to support relatives/caregivers of individuals with borderline personality disorder and related emotion dysregulation. Family Connections is primarily caregiver-focused, whereas STEPPS is a patient-directed, systems-based programme that combines psychoeducation, emotion-management skills, and behavior-management/problem-solving skills while also involving family members or other support-system members in reinforcing these skills. Because our trial aimed to augment a DBT-informed skills intervention for hospitalized adolescents themselves, while also strengthening family-supported generalization of coping strategies, STEPPS was considered a more appropriate adjunctive module for this specific clinical and service context. And the results from its application in an intervention addressing self-harm behaviors among Spanish prison inmates indicate a reduction in such behaviors (13).

Therefore, in the present study, STEPPS was not conceptualized as a substitute for DBT, but as an adjunct designed to extend and reinforce DBT skills in a more structured and system-oriented format.

Although DBT and STEPPS share content related to emotion regulation, this overlap may represent therapeutic reinforcement rather than redundancy. For adolescents with NSSI, self-injury is often maintained not only by acute affective instability, but also by limited coping repertoires, poor transfer of skills into daily life, and invalidating interpersonal environments (14). Under this framework, DBT provides the core acceptance–change model, including mindfulness, distress tolerance, emotion regulation, and interpersonal effectiveness, whereas STEPPS may strengthen skills consolidation through repeated practice, explicit behavioral problem-solving, crisis-response rehearsal, and family-supported generalization of adaptive coping. We therefore hypothesized that a DBT–STEPPS model would be novel and clinically feasible because it combines an evidence-based core treatment with a structured adjunctive programme targeting both intrapersonal dysregulation and the interpersonal context of NSSI. We further hypothesized that this combined approach would yield greater reductions in NSSI than DBT alone.

Although reduction of NSSI was the primary clinical objective of this trial, we also considered depressive symptoms to be an important secondary outcome. This is because adolescent NSSI commonly co-occurs with depression and anxiety (15), and that these conditions appear to share several maintaining mechanisms, particularly emotion dysregulation, maladaptive cognitive coping, behavioral avoidance, and interpersonal invalidation (16). Accordingly, an intervention that improves emotional awareness, distress tolerance, adaptive coping, and family-supported problem solving may not only reduce self-injury, but also yield secondary improvements in depressive symptoms. In this framework, we did not hypothesize that DBT-STEPPS would treat depression as an isolated syndrome per se; rather, we hypothesized that depressive symptoms might decrease as a downstream consequence of targeting the broader affective and interpersonal processes that sustain NSSI.

## Methods

### Study design

This study is a single-blind, randomized controlled trial in which patients were randomly and evenly allocated to the Control Group, Intervention Group A, and Intervention Group B in a 1:1:1 ratio. This study was approved by the Ethics Committee of the Second Affiliated Hospital of Xinxiang Medical University (XYEFYLL-(Research)-2021-09-1). A research assistant, unaware of the group assignments, generated a randomized sequence of numbers (from 001 to 192) using the Research Randomizer website.^1^ Group information was placed in opaque envelopes, subsequently sealed, and only accessible to this assistant during the random allocation process. Participants were recruited in a natural, unpredictable sequence. Following screening and confirmation of eligibility, the intervention implementer consulted the assistant regarding group assignment. This study conducted a blind experiment on the subjects. All the interventions were carried out under the guise of “daily activities.” Due to the nature of the study design, both the intervention implementer and the patient themselves were aware of the group allocation, whereas the scale assessors remained unaware of the specific group details.

### Participants

We recruited study subjects for adolescent NSSI patients who were hospitalized from January 2024 to August 2024 in the Second Affiliated Hospital of Henan Medical University. All patients participating in the study provided written informed consent, and for those younger than 16, consent was obtained from their parents or legal guardians.

Inclusion criteria: (1) Meets diagnostic criteria for depressive disorder according to the Diagnostic and Statistical Manual of Mental Disorders, Fifth Edition (DSM-5; without psychotic symptoms); assesses presence of non-suicidal self-injury (NSSI) behavior per DSM-5 criteria, confirming NSSI with fulfilment of either frequency criterion: ① ≥3 instances of NSSI within the past 6 months, with at least one occurrence within the past month; ② ≥5 episodes within the past year, with at least 1 episode within the past month. (2) Hamilton Depression Rating Scale (HAMD-17) score ≥18 and ≤24, with item 3 scored as 0. (3) Male or female adolescents aged 12–18 years; (4) Capable of independently completing questionnaire assessments.

Exclusion criteria: (1) Presence of other major mental disorders, such as schizophrenia spectrum disorders, bipolar affective disorder, autism spectrum disorders, or intellectual developmental disorders; (2) Concurrent severe, unstable somatic illness likely to significantly impact mental state or assessment; (4) Current moderate to high suicide risk; (5) Recent systematic treatment within the past month involving dialectical behavior therapy or cognitive emotive and problem-solving systems training.

We estimated the sample size according to the NSSI behavioral scores before and after the pre-experimental treatment about the formula: $n=\psi^{2}(\sum s_{i}^{2}/g)/[\sum ({\bar{x}}_{t}-\bar{x})/(g-1)]$ (*n* is the sample size of each group, ${\bar{X}}_{t}$ , $S_{i}$ is the mean and standard deviation of each group, *g* is the number of groups, *α* is taken to be 0.05, *β* is taken to be 0.02, as per the table *ψ* is 2.17). It can be calculated that *n* = 58. Considering 10% loss of visit rate, 192 cases were finally included.

One of the members of this research team identified potentially eligible participants from the hospital’s inpatient medical record system (HIS system) records based on inclusion and exclusion criteria. Following admission, the assistant introduced the investigators and the primary research objectives to potentially eligible paediatric patients and their families to establish trust and obtain consent from both the patient and their guardian. Furthermore, participants may opt out of the study at any time during its course.

No patients or members of the public were involved in the design, conduct or reporting of this clinical trial.

### Intervention

The Control Group received an eight-week standardized comprehensive intervention comprising standardized psychiatric medication treatment, foundational mental health education (weekly group lectures covering stress management, healthy lifestyle practices, etc.), and psychological counselling (30-min weekly individual supportive sessions conducted by mental health practitioners). The Intervention Group A comprised the control group’s intervention content alongside DBT intervention. Intervention Group B comprised the interventions received by Group A alongside STEPPS intervention. Both DBT and STEPPS interventions lasted for 8 weeks, with sessions held once weekly for 45 to 60 min. Details are as follows:

### Form an intervention team

The personnel who implemented the intervention in this study consisted of three psychiatrists, six psychiatric rehabilitation therapists (who had been authorized by the hospital’s Committee for the Management of Clinical Application of Medical Technology), and six psychiatric nurses (who had been certified as China’s second-level counsellors). The psychiatrists were responsible for disease diagnosis, medication, and scale assessment of the study subjects. The psychiatric nurses were responsible for the collection and organization of records of the intervention process. Rehabilitation therapists were responsible for implementing the intervention, documenting the intervention process. Psychiatric rehabilitation therapists provide standardized guidance and training to the team, coordinating with psychiatric nurses to deliver patient health education. Finally, all members of the rehabilitation team completed training in DBT combined with STEPPS to ensure the quality and consistency of rehabilitation services.

### Develop the intervention plan

The intervention programme was developed based on the book Dialectical Behavior Therapy (17) reviewing research on adolescent non-suicidal self-injury, depression, and related interventions over the past 5 years. It focused on analyzing the evidence-based efficacy and applicability of both DBT and STEPPS methodologies. Building upon this foundation, the research team designed an intervention framework integrating core DBT skills (mindfulness, distress tolerance, emotion regulation, interpersonal effectiveness) with STEPPS modules for emotion management and problem-solving.

To differentiate the two programmes, we predefined distinct therapeutic functions for DBT and STEPPS based on their original treatment models. The DBT component was derived from the standard DBT skills model and was used as the core intervention for intrapersonal self-regulation, with primary emphasis on mindfulness, distress tolerance, emotion regulation, and interpersonal effectiveness. In contrast, the STEPPS component was derived from the manualised systems-based STEPPS model and was used as an adjunctive intervention focused on psychoeducation, emotional-intensity monitoring, structured problem-solving, behavior management, and involvement of family/support-system members. Thus, DBT primarily targeted acquisition of core coping skills, whereas STEPPS primarily targeted consolidation, rehearsal, and generalisation of those skills in everyday and family contexts. Where similar topics were addressed in both programmes, they were intentionally differentiated by therapeutic level and purpose rather than taught as duplicate material. For example, DBT sessions focused on teaching and practising core skills within the acceptance–change framework, whereas STEPPS sessions focused on translating these skills into simplified, structured, and family-reinforced action plans for emotional crises and daily problem solving. Operationally, DBT strategies were classified as primary skills-acquisition strategies, whereas STEPPS strategies were classified as secondary systems-oriented reinforcement strategies.

To enhance the programme’s professional rigor, multiple senior experts in DBT, STEPPS, and adolescent psychotherapy were invited to participate in deliberations. Following two rounds of expert consultation by correspondence, incorporating specialist feedback, a localized intervention programme was finalized. We initially invited 10 specialists (three psychiatrists, three mental health rehabilitation therapists, and four psychotherapists). Ultimately, eight completed all rounds, yielding a response rate of 80%. Kendall’s *W* = 0.68 (*p* < 0.01), Cr = 0.82.

### Intervention content for Intervention Group A

The Control Group received routine inpatient care, including pharmacotherapy, foundational mental health education, and weekly supportive individual counselling. Intervention Group A received the same routine inpatient care plus an adapted DBT-informed skills-based group intervention. This intervention employs a ‘progressive training of core skills modules’ approach, unfolding across six steps. Sessions are held weekly, each lasting 45–60 min. See Table 1 for details.

**Table 1:** Elements of the DBT intervention in this study.

Time	Name	Element
Week 1	Establishing therapeutic alliances and evaluating patients	Through the first interview, a therapeutic relationship can be established with the patient; the patient’s behavioral characteristics of NSSI are systematically assessed; and the patient is given a clear picture of the four core intervention goals of positive thinking training, pain tolerance, emotion regulation, and interpersonal efficacy.
Week 2	Positive thinking skills training	Basic Perception Training: Enhance present moment concentration through breath monitoring (guided somatic awareness), auditory focus (relaxation through musical imagery), and visual observation (detailed descriptions of clay sculptures). Cognitive Reconstruction Training: Use of divergent thinking techniques (e.g., role-play imagery) to break cognitive rigidity, combined with cognitive diary entries to achieve non-judgmental self-awareness.
Week 3–4	Pain tolerance training	Physiological Module: Enhancement of stress threshold through somatic interventions such as cold water tolerance and progressive muscle relaxation. Psychological coping module: establish alternative coping strategies through self-acceptance training (guiding patients to talk about stressful events they have experienced and how they felt, instructing them to face the facts openly, applying positive thinking skills to dissolve them, and realising self-acceptance) and supplemented with five-sense soothing (reading, music, etc.).
Week 5–6	Emotional regulation training	Emotion log analysis: instructing patients to record emotionally triggering events and correcting cognitive biases through reality-testing techniques (e.g., two-column evidence comparisons). Behavioral Activation Intervention: Develop a personalised positive activity plan to enhance positive emotional experiences through behavioral experiments (art creation, volunteering, etc.).
Week 7	Interpersonal effectiveness training	DEAR MAN Technique: Train core communication skills such as Describe, Express, and Assert in simulated social situations. Relationship Maintenance Enhancement: Practice the GIVE (Gentleness, Empathy, Feedback) and FAST (Self-Respect, Moderate Compromise) dual-module techniques through role-playing.
Week 8	Treatment consolidation and relapse prevention	Summarise examples of skills application, develop personalised maintenance plans, and strengthen self-monitoring skills. Weekly 60-min group therapy sessions combined with 15 min of skill practice each day. The therapist provides 20 min of individualised feedback each week.

The DBT-based intervention used in this study was not intended to represent a full standard comprehensive DBT-A programme. Rather, it focused primarily on the DBT skills-training component, including mindfulness, distress tolerance, emotion regulation, and interpersonal effectiveness, delivered in weekly face-to-face group sessions.

### Intervention content for Intervention Group B

Intervention Group B received the same routine inpatient care plus the adapted DBT-informed skills-based group intervention and the STEPPS programme. The DBT component mirrored that of Intervention Group A. The STEPPS intervention comprised 8 weeks of in-person group sessions conducted by rehabilitation therapists. Sessions occurred twice weekly (one DBT session and one STEPPS session), each lasting 45–60 min. The intervention framework comprised five progressive modules, detailed in Table 2.

**Table 2:** Elements of the STEPPS intervention in this study.

Time	Name	Element
Week 1	Initial assessment and relationship building	The therapist established a therapeutic alliance with the patient through self-introduction and systematically assessed the patient’s behavioral characteristics of NSSI, clarified the principles of STEPPS intervention to the patient, and worked with the patient to formulate the three core goals: to strengthen emotional management, to enhance behavioral regulation, and to promote family synergy.
Week 2	Deepening psychological education	The therapist explained the NSSI emotion-behavior linkage model to the patient to deepen the patient’s understanding of NSSI; at the same time, a family handbook was distributed to guide psychoeducation for the patient and his family, to make them realize that the NSSI behavior is not the only way to solve the problem, and to instruct the family to adopt a non-judgemental mode of communication, to help the patient to cognitively perceive the alternative coping strategies.
Week 3–4	Intensive emotional management	The goals were established as negative emotion recognition, understanding, and adjustment, and reduction of self-injurious behaviors driven by adverse emotions. Emotion regulation skills were divided into 2 categories. Patients were instructed on emotional response change training in week 3, using a diary to record relevant events and their emotional course, and were guided to check the facts and assess the reasonableness of their emotional responses. If the event was consistent with the emotional response, no adjustment was needed. If the event is not consistent with the emotion, the patient is instructed to adopt the opposite emotion to cope with it, such as adopting a mild emotion to deal with something that makes them feel angry. In week 4, patients were instructed to develop positive emotions, cultivate hobbies and interests, establish a list of positive experiences, and formulate an implementation plan, such as listening to music, learning calligraphy, reading books, and participating in public welfare activities. Gradually increase the frequency or difficulty over time.
Week 5–7	Behavioral reshaping training	The objectives were established as: enhancing problem-solving skills, improving interpersonal communication skills, and reducing NSSI behavior. Behavior management skills were divided into 3 major categories. Patients were instructed in week 5 to use structured problem-solving training, which was implemented in the order of clarifying the problem → listing the options → evaluating the pros and cons → implementing the options. Patients and their families were instructed in interpersonal communication skills training in week 6, focused on structured communication rehearsal and family-supported problem solving. In the 7th week, the patients and their families were instructed to simulate the crisis response training, which was implemented in the order of early warning → emergency response → security measures environment.
Week 8	Terminal evaluation and consolidation	Multi-dimensional assessment tools are used to quantify the effectiveness of the intervention and develop a personalised maintenance plan. The programme is designed in a modular way to achieve a step-by-step progression of skills training, and the concept of family system support is integrated throughout the programme. The Emotion Management Module complements DBT, while the Behavior Management Module focuses on the development of real-life problem-solving skills.

### Observation indicators

#### NSSI behavior

The Adolescent Non-suicidal Self-injury Assessment Questionnaire (ANSAQ) developed by Yuhui Wan et al. (18) was used for assessment. The scale is divided into 2 sub-questionnaires, the Adolescent NSSI Behavioral and Functional. The Behavioral Questionnaire includes NSSI behaviors without significant tissue damage (7 items) and NSSI behaviors with significant tissue damage (5 items), with 2 dimensions and 12 entries. Each entry was scored on a 5-point Likert scale (0–4), with the higher the score, the more severe the NSSI behavior. The Functional Questionnaire included self-interested socialization, negative self-reinforcement and emotional expression, with 3 dimensions and 19 entries. Each item employs a five-point Likert scale (0–4 points), with higher scores indicating greater reliance on NSSI as a coping mechanism. The Cronbach’s coefficients of the two sub-questionnaires in this questionnaire were 0.921 and 0.905 that order.

#### Emotional regulation ability

The Cognitive Emotion Regulation Questionnaire-Chinese Version (CERQ-C) developed by Xiongzhao Zhu et al. (19) was used for the assessment. The scale includes 9 dimensions and 36 items, including self-blame, rumination and catastrophizing. Each entry was rated on a 5-point Likert scale (1–5). Acceptance, positive refocusing, positive reappraisal, refocusing on the plan, and rational analysis, which are 5 dimensions and 20 entries, were summarized as adaptive adjustment strategies. Self-blame, contemplation, blaming others and catastrophizing, which are 4 dimensions and 16 entries, were summarized as non-adaptive regulation strategies. Higher scores suggest that patients will favor a certain emotion regulation strategy when faced with a stressful event, with a Cronbach’s coefficient of 0.810 for this scale.

#### Severity of illness

The Self-Rating Anxiety Scale (SAS) (20) and The Self-Rating Depression Scale (SDS) (21) were used for assessment. Each of the two scales has 20 items, and each assessment item is rated on a Likert 4 scale (1–4), with a total score of 80. Crude scores were calculated on a scale of 1:1.25, and the critical values of SAS and SDS were 50 and 53 in that order. Higher scores indicated a more severe state of anxiety and depression, and the Cronbach’s coefficients for the SAS and SDS scales were 0.912 and 0.920, respectively.

Questionnaire selection was guided by the prespecified outcome domains of the trial rather than by allegiance to a single psychotherapy model. Because the present study aimed to evaluate changes in NSSI behavior/function, emotion regulation, and anxiety/depressive symptoms, we selected instruments that directly assessed these clinical domains.

### Quality control

All doctors, nurses, and rehabilitation therapists participating in the study were certified as physicians or nurses or psychotherapists or counsellors. Rehabilitation therapists received skills training and obtained hospital-certified rehabilitation therapy qualification, and all of them uniformly received special DBT and STEPPS training. The investigators received uniform training and were familiar with the content of the scales, how to fill them out, and the precautions to be taken. A total of 192 questionnaires were distributed in this study. All questionnaires were returned and were found to be valid, achieving a 100.00% response rate. Two persons checked the questionnaires and processed the questionnaire data to ensure the reliability, completeness and accuracy of data entry. The person in charge shall observe the entire intervention process to ensure its quality.

Adverse events include negative reactions, psychological distress, social impacts, or other unforeseen consequences related to the intervention. The research team will monitor and collect adverse event data throughout the study. Participants will be informed to report any adverse reactions confidentially at any time. Data will be collected via face-to-face interviews, questionnaires, and phone calls to ensure comprehensive identification of potential harms. Clinical teams will assess whether adverse events are intervention-related and classify their severity.

### Statistical methods

Data analysis was conducted using SPSS 22.0 software. Quantitative data meeting normality criteria confirmed by Shapiro–Wilk test were presented as mean ± standard deviation ( $\bar{x}\pm s$ ). Intergroup comparisons employed one-way ANOVA, with pairwise post-hoc comparisons using Bonferroni correction. Non-normally distributed quantitative data were presented as median (interquartile range) [M (P25, P75)]. Intergroup comparisons employed the Kruskal–Wallis *H* test. Categorical data were expressed as case numbers (percentages) [*n* (%)]. Intergroup comparisons utilized the *χ* (2) test or Fisher’s exact test. A *p* value < 0.05 was considered statistically significant. In addition to significance testing, effect sizes were reported to reflect the magnitude of between-group differences. Analyses were performed on the intention-to-treat (ITT) population, defined as all randomized participants, analyzed according to the group to which they were randomized. Since this study was integrated into routine clinical practice, no interim analyses were performed and no stopping guidelines were established. In addition, there were no missing data in this study. No additional analyses (including subgroup or sensitivity analyses) were performed in this study.

## Results

Since the intervention measures of this study were integrated into the daily work, no participants withdrew halfway, and no participants suffered any injuries or accidents. Therefore, the number of participants in each group for data analysis was 64. The intervention measures were implemented by the staff. Under the hospital’s working system and the supervision of the leaders, the quality of the work was reliable, so there was no need to measure the accuracy. The detailed results are as follows.

### Baseline information

Comparison of gender, age and duration of disease in the three groups did not differ significantly for comparison (*p* > 0.05), see Table 3 for details.

**Table 3:** Baseline information for the three groups.

Group	Sex (male/female) (*n*)	Age (years)	Medical history (years)
Control Group (*n* = 64)	20/44	15.06 ± 1.42	1.75 ± 0.51
Intervention Group A (*n* = 64)	26/38	15.13 ± 1.37	1.80 ± 0.52
Intervention Group B (*n* = 64)	22/42	14.98 ± 1.40	1.73 ± 0.49
*F/χ^2^*	1.275	0.185	0.324
*p*	0.529	0.813	0.724

#### NSSI behavior

Compared with pre-intervention levels, all three intervention groups demonstrated reductions in adolescent NSSI and functional behavior scores post-intervention (*p* < 0.05). Comparisons of adolescent NSSI behaviors and functional scores across the three intervention groups revealed statistically significant differences (*p* < 0.05). Intervention Group B exhibited lower NSSI behaviors and functional scores than both Intervention Group A and the control group. Observation revealed that Intervention Group A’s NSSI behaviors and functional scores were lower than those of the control group (*p* < 0.05). See Table 4 for details.

**Table 4:** Results of NSSI behavioral and functional scores in three groups ( $\bar{x}\pm s$ ).

Group	Behavioral score (points)		Functional score (points)	
	Pre-intervention	Post-intervention	Pre-intervention	Post-intervention
Control group (*n* = 64)	36.24 ± 5.26	25.72 ± 4.13	46.04 ± 8.19	34.17 ± 6.80
Intervention Group A (*n* = 64)	35.89 ± 5.17	19.47 ± 3.27^a^	46.21 ± 8.20	28.71 ± 5.43^a^
Intervention Group B (*n* = 64)	36.37 ± 5.29	16.03 ± 2.84^a^^,^^b^	46.95 ± 8.54	21.41 ± 4.36^a^^,^^b^
*F*	0.144	129.368	0.217	83.068
*p*	0.867	0.000	0.805	0.000
*η^2^*	/	0.578	/	0.468

Comparison with control group.

a *p* < 0.05; comparison with Intervention Group A.

b *p* < 0.05.

#### Emotional regulation ability

Compared with pre-intervention, adaptive regulatory strategies in CERQ-C increased and non-adaptive regulatory strategies decreased in all three groups after intervention (*p* < 0.05). Comparison of adaptive regulation strategy and non-adaptive regulation strategy scores in CERQ-C after intervention in the three groups, the difference was statistically significant (*p* < 0.05). The adaptive regulation strategy scores in the Intervention Group B were higher than those in the Intervention Group A and control group, and the non-adaptive regulation strategy scores in the Intervention Group B were lower than those in the Intervention Group A and control group (*p* < 0.05). Adaptive conditioning strategy scores were higher and non-adaptive conditioning strategy scores were lower in the Intervention Group A than in the control group (*p* < 0.05). See Table 5 for details.

**Table 5:** Results of the three groups of emotion regulation scores ( $\bar{x}\pm s$ ).

Group	Adaptive adjustment strategy score (points)		Non-adaptive adjustment strategy score (points)	
	Pre-intervention	Post-intervention	Pre-intervention	Post-intervention
Control Group (*n* = 64)	59.01 ± 9.76	66.45 ± 5.12	54.31 ± 8.84	43.74 ± 5.51
Intervention Group A (*n* = 64)	58.87 ± 9.62	72.41 ± 4.01^a^	54.18 ± 8.67	39.45 ± 5.04^a^
Intervention Group B (*n* = 64)	59.06 ± 9.81	79.42 ± 3.56^a^^,^^b^	54.40 ± 8.35	35.41 ± 4.65^a^^,^^b^
*F*	0.007	147.217	0.011	43.054
*p*	0.993	0.000	0.990	0.000
*η^2^*	/	0.609	/	0.313

Comparison with control group.

a *p* < 0.05; comparison with Intervention Group A.

b *p* < 0.05.

#### Severity of illness

Compared with the pre-intervention period, the SAS and SDS scores of the three groups decreased after the intervention (*p* < 0.05). SAS and SDS scores were compared among the three groups after intervention (*p* < 0.05). The SAS and SDS scores of Intervention Group B were lower than those of Intervention Group A and Control Group (*p* < 0.05). The SAS and SDS scores of the Intervention Group A were lower than those of the Control Group (*p* < 0.05). See Table 6 for details.

**Table 6:** Anxiety and depression status scores in the three groups ( $\bar{x}\pm s$ ).

Group	SAS (points)		SDS (points)	
	Pre-intervention	Post-intervention	Pre-intervention	Post-intervention
Control Group (*n* = 64)	58.06 ± 6.15	50.45 ± 5.02	55.47 ± 5.76	48.92 ± 4.72
Intervention Group A (*n* = 64)	58.11 ± 6.20	45.27 ± 4.61^a^	55.81 ± 5.68	43.76 ± 4.25^a^
Intervention Group B (*n* = 64)	58.22 ± 6.07	41.28 ± 4.08^a^^,^^b^	55.64 ± 5.71	40.02 ± 3.99^a^^,^^b^
*F*	0.011	64.326	0.057	68.153
*p*	0.989	0.000	0.945	0.000
*η* ^2^	/	0.405	/	0.419

Comparison with control group.

a *p* < 0.05; comparison with intervention Group A.

b *p* < 0.05.

## Discussion

The emergence and progression of adolescent NSSI behavior stems from the intricate interplay of neurobiological and socio-environmental factors. On one hand, alterations such as dysfunction of the hypothalamic–pituitary–adrenal (HPA) axis and diminished endogenous opioid levels constitute its underlying biological foundation (22, 23); on the other, social discrimination, detrimental parenting patterns, and various stressful life events persistently act as significant environmental triggers. Consequently, adolescents with NSSI frequently employ physical self-harm methods such as head banging, cutting, scratching, or burning to cope with overwhelming negative emotions. This not only exacerbates psychological distress but may also lead to persistent physical injury and diminished social functioning.

Clinically, traditional interventions (such as supportive psychotherapy alone or cognitive behavioral therapy) may reduce self-harming behaviors in the short term, yet they commonly exhibit limitations in efficacy and struggle to achieve lasting consolidation, leaving considerable scope for improvement in overall effectiveness.

Existing research suggests that DBT can be productively augmented by additional intervention modules when these modules target clinically relevant mechanisms that are not fully addressed by the core programme alone. For example, in a pilot randomized trial among suicidal and self-injuring women with borderline personality disorder and PTSD, adding the DBT Prolonged Exposure protocol to standard DBT was feasible, acceptable, and associated with larger improvements than DBT alone across PTSD and several secondary outcomes (24). Likewise, DBT-PTSD represents a structured integration of DBT principles with trauma-focused treatment components and has demonstrated efficacy in complex PTSD. Although these interventions target different clinical populations and do not test the present DBT-STEPPS model directly, they support the broader therapeutic principle that modular augmentation of DBT may be beneficial when the added component addresses a complementary domain of psychopathology or skill generalization. Therefore, the present study should be understood as testing this broader integrative principle in a new target population rather than confirming a previously established combined protocol.

### DBT combined with STEPPS intervention helps reduce self-injurious behavior in adolescents with NSSI

This study employed the Adolescent Non-Suicidal Self-Injury Assessment Questionnaire, developed in recent years for the domestic adolescent population, to conduct a multidimensional assessment of NSSI behaviors. This instrument comprehensively examines both the severity of the behavior (whether it resulted in significant tissue damage) and its functional aspects (such as motives including self-serving socialization, self-deactivation, and emotional expression). It provides a relatively thorough reflection of the impact of intervention measures on adolescent self-injury behaviors. Results indicated that compared with both the Control Group and Intervention Group A, Intervention Group B demonstrated significantly lower scores on both the severity and functional dimensions of NSSI behavior. This suggests that the combined DBT and STEPPS intervention may possess broader applicability for addressing NSSI behaviors exhibiting diverse manifestations and motivations.

Specific analysis (25): Firstly, DBT emphasizes the dialectical balance between acceptance and change. Through mindfulness training, patients learn to experience the present moment without judgement, consciously feeling their current emotions and observing events unfolding around them without distorting reality. Two seemingly contradictory things may both be valid; every event and person is interconnected in some way. Change is the only constant, and it is inherently interactive. The child should adopt effective methods to resolve events that have already occurred.

Secondly, through pain tolerance training, patients learn that suffering is an inherent part of life, impossible to evade or eliminate. They may grant themselves a moment’s respite or take a deep breath to create a buffer zone, thereby avoiding impulsive self-harm as a coping mechanism. When overwhelmed by intense emotions and tempted to resort to self-harm, they may employ techniques such as splashing cold water on their face, holding ice cubes, or engaging in sensory distractions—like reading, listening to music, taking a walk, or conversing—to swiftly reduce suicidal thoughts and impulses. Through emotional regulation training, patients learn to identify, understand and manage negative emotions. This involves journaling, cultivating hobbies, and establishing a positive experiences checklist to practice altering emotional responses and nurturing positive feelings. This approach helps patients develop positive emotions and engage more fully in learning and daily activities where they excel. Interpersonal communication skills training further enhances their ability to interact effectively with others.

Through interpersonal communication skills training, patients’ social interaction abilities can be enhanced. Techniques such as DEAR MAN, GIVE, and FAST can specifically improve patients’ communication skills in various areas, including treating others as equals, being gentle and sincere, and engaging in friendly discussions. Moreover, the DEAR MAN technique was employed in both DBT and STEPPS, potentially yielding superior outcomes in Intervention Group B due to the skill’s “dose effect” or synergistic reinforcement. Receiving similar concepts from slightly different perspectives—the individual-centred approach in dialectical behavior therapy versus the family-involved approach in STEPPS—and at a higher frequency, may have facilitated deeper learning and knowledge transfer. STEPPS integrates emotional regulation training with cognitive behavioral components, placing greater emphasis on recognizing emotional fluctuations and structured problem-solving. By cultivating emotional anticipation and problem-solving abilities, it helps adolescent patients understand their condition and encourages proactive treatment engagement. This enables young people to pre-emptively identify high-risk situations potentially triggering self-harm, develop coping strategies, and strengthen control over self-harm impulses.

Intervention Group B, in addition to employing DBT strategies, integrated STEPPS and distributed family manuals to guide psychological education for patients and their families. This approach helped patients recognize that NSSI was not the sole solution, while instructing family members to reduce accusatory language and adopt warmer, more empathetic communication styles. Consequently, this effectively reduced the incidence of NSSI among patients. Research by Liu et al. (26) confirmed that DBT contributes to reducing self-harm behaviors among adolescent NSSI patients during the six-month intervention period (42.2% vs. 67.4%). Evidence from Victor et al. (27) indicates that the quality of adolescent-parent relationships serves as a longitudinal predictor of self-harm behaviors, while promoting family member involvement through STEPPS-like group interventions helps reduce such behaviors. These findings align with the conclusions of the present study, further supporting the feasibility and efficacy of combined DBT and STEPPS interventions in preventing adolescent NSSI.

### DBT combined with STEPPS intervention helps adolescents with NSSI to regulate mood and improve negative emotions

The occurrence of NSSI behaviors is strongly associated with difficulties in emotion regulation and coping with negative life events. A non-randomized pilot study (28) showed that independent influences on the development of NSSI behavior were poor functional coping and emotional dysregulation, and that DBT may help people with NSSI to regulate negative emotions. Another mediating effect (29) confirmed that the etiology of NSSI in adolescents is related to childhood maltreatment, and that difficulties in emotion regulation and depressive symptoms may play a mediating role, especially in the case of difficulties in emotion regulation. Thus, improving their functional coping and emotion regulation skills is a major intervention factor to reduce NSSI behaviors in adolescents with NSSI.

The findings of this study indicate that participants in Intervention Group B demonstrated significantly higher scores across multiple adaptive emotional strategies (such as acceptance, positive refocusing, positive reappraisal, refocusing plans, and rational analysis) compared to both the control group and Intervention Group A. Conversely, their scores for maladaptive strategies (such as self-blame, rumination, blaming others, and catastrophizing) were significantly lower. Furthermore, they exhibited a more pronounced reduction in SAS and SDS scores (*p* < 0.05). This suggests that the combined intervention may have effectively altered patients’ internal patterns of coping with negative emotions. Prior to receiving the intervention, patients often found themselves trapped in a vicious cycle: negative emotions triggered ineffective coping strategies (such as rumination or catastrophic thinking), which not only failed to alleviate distress but exacerbated emotional deterioration, thereby reinforcing dependence on maladaptive approaches. Through DBT and STEPPS interventions, patients learn to proactively employ adaptive strategies when emotional distress arises, gradually establishing a virtuous cycle of ‘emotional awareness–strategy activation–emotional relief–confidence enhancement’. This process not only directly alleviates negative emotions such as anxiety and depression but also enhances patients’ sense of efficacy in self-managing their emotions, thereby promoting the automatic formation of healthy coping patterns. This is in line with the results of an uncontrolled open trial conducted by Bjureberg et al. (30) scholars, which confirmed that the application of individual therapy for emotion regulation in adolescents with NSSI was somewhat similar in terms of the frequency of NSSI, difficulties in emotion regulation, and some benefit in overall functioning.

Therefore, from the perspective of intervention mechanisms, both DBT and STEPPS employ complementary strategies to regulate emotions and alleviate anxiety and depressive symptoms. For instance, mindfulness, distress tolerance skills, and emotion regulation training involve rehabilitation therapists explaining the causes of negative emotions such as anxiety, depression, and anger—that is, the thoughts and reactions following an event, rather than a reflection of the event’s reality. By validating emotional responses, these approaches facilitate rational resolution. Within the present model, DBT contributed the core acceptance-and-change framework by teaching mindfulness, distress tolerance, emotion regulation, and interpersonal effectiveness, whereas STEPPS provided a more repetitive and structured adjunct through psychoeducation, behavioral problem-solving, crisis planning, and family involvement. Together, these components may reduce emotional helplessness, strengthen perceived coping efficacy, and increase the use of adaptive responses to negative affect. From this perspective, the improvement in depression observed in the combined group should be interpreted as a secondary effect of modifying transdiagnostic mechanisms shared by NSSI and depression, rather than as evidence that depression itself was the primary therapeutic focus. Because the present study did not include mediation analyses, this mechanistic interpretation remains hypothetical and should be tested in future studies.

The potential benefits of the present combined intervention may be considered at both the individual and clinical-practice levels. At the individual level, a model that combines DBT-based self-regulation skills with the structured psychoeducation, problem solving, and family/system involvement of STEPPS may help adolescents replace self-injury with more adaptive coping responses, improve emotional awareness and self-efficacy, and reduce associated anxiety and depressive symptoms (9, 25). The expected benefit, therefore, is not only fewer self-injurious acts, but also improved capacity to manage distress without relying on self-harm. At the clinical-practice level, the proposed combination may be relevant because it is structured, manual-guided, and potentially compatible with routine adolescent psychiatric care. In the present study, the intervention was delivered by a trained multidisciplinary team within routine inpatient services, suggesting that this model may be implementable in real-world clinical settings. In addition, STEPPS was originally developed as a systems-based, manualised adjunctive programme that is comparatively straightforward to deliver and explicitly incorporates family or other support-system members. This may increase the practical transfer of coping skills from the therapy setting to the ward, the home environment, and post-discharge daily life.

However, these implications should be interpreted cautiously. The present study was single-centre, relied mainly on self-report outcomes, and did not assess long-term maintenance, implementation outcomes, or cost-effectiveness. Accordingly, the clinical significance of this model should be regarded as preliminary and in need of replication in larger, multi-center studies.

Besides, Sex/gender and age are important clinical variables in adolescent NSSI. Previous studies have shown that the prevalence and clinical presentation of NSSI vary across developmental stages and between males and females (31). However, evidence that DBT-based treatment effects differ systematically by sex or age remains limited. In a meta-analysis of DBT for adolescents, neither mean age nor the proportion of female participants significantly moderated treatment effects on self-harm or suicidal ideation (32). Therefore, although these variables are clinically relevant, the present study cannot determine whether the efficacy of the DBT-STEPPS model differs across sex or age groups.

### Limitations

This study is a single-centre and small-sample study, which may result in biased findings. This study did not conduct follow-up assessments of outcomes after the intervention concluded, making it impossible to determine the long-term efficacy of the combined DBT and STEPPS intervention or the stability of behavioral changes. Future follow-up research is required to validate its long-term benefits. In addition, although sex/gender and age may influence the clinical presentation of adolescent NSSI, this trial did not prespecify subgroup analyses by sex or age and was not powered to detect interaction effects. Therefore, we could not draw reliable conclusions regarding differential treatment efficiency across these subgroups. Future studies with larger samples and prospectively planned subgroup or interaction analyses are needed.

The primary outcome measures in this study were derived solely from patient self-report questionnaires, without incorporating objective physiological indicators or third-party behavioral observations, potentially introducing social desirability bias. The intervention implemented in this study did not incorporate other modules of DBT or STEPPS. Future research may enrich the intervention content to validate the generalizability of DBT and STEPPS. In this study, the intervention was only observed by the principal investigator during delivery to ensure quality, but therapist adherence was not assessed. Future research should pay attention to evaluating therapist adherence.

We did not include a DBT-specific process measure such as the DBT-WCCL; therefore, although clinical outcomes improved, we could not directly examine whether changes in DBT skills use mediated these effects.

The present study did not include a formal health-economic evaluation, cost-effectiveness was not assessed and should be examined in future research.

In addition, cultural and socioeconomic variation was not formally assessed in this study. Although participants were recruited from a single regional inpatient setting, we did not collect variables such as family income, parental education, ethnicity, or urban–rural background. Therefore, the generalisability of the findings across different cultural and economic contexts remains uncertain.

## Conclusion

The use of DBT combined with STEPPS intervention in adolescent NSSI patients can decrease their self-injurious behaviors and improve their emotional regulation, which is highly important for enhancing adolescent mental health and preventing depression relapse. The findings also offer preliminary insights into the clinical pathways, such as emotion regulation, that may be targeted to reduce NSSI behaviors. This study provides a valuable perspective for developing and optimizing psychotherapeutic approaches for adolescent NSSI, which can be promoted and implemented in psychiatric wards.

### What is already known

- Dialectical Behavior Therapy (DBT) has demonstrated effectiveness in reducing non-suicidal self-injury (NSSI) behaviors and improving emotional regulation in adolescents through its structured skills training modules.
- Systems Training for Emotional Predictability and Problem Solving (STEPPS) has shown promise in addressing self-harm behaviors, particularly in populations with borderline personality traits, through its emphasis on emotion management and family involvement.
- Current conventional psychological interventions for adolescent NSSI, including standalone supportive psychotherapy or cognitive behavioral therapy, often show limited efficacy due to their inability to dynamically adapt to individual patient needs and symptom severity.

### What this paper adds

- Combined DBT and STEPPS intervention demonstrates superior efficacy compared to DBT alone or conventional treatment in reducing NSSI behaviors and improving emotion regulation among adolescents with NSSI.
- The integrated intervention significantly enhances adaptive emotion regulation strategies (acceptance, positive refocusing, positive reappraisal) while reducing maladaptive strategies (self-blame, rumination, catastrophizing) more effectively than monotherapy.
- The combination approach yields greater reductions in anxiety and depression symptoms compared to DBT alone, suggesting synergistic effects when targeting both individual skill development and family-involved psychoeducation.

## Data Availability

The raw data supporting the conclusions of this article will be made available by the authors, without undue reservation.

## Footnotes

1
https://www.randomizer.org/
